# Czech Honeydew Honeys—A Potential Source of Local Medical Honey with Strong Antimicrobial Activity

**DOI:** 10.3390/ph17070840

**Published:** 2024-06-27

**Authors:** Ludovit Pudelka, Radek Sleha, Sylva Janovska, Vera Radochova, Pavel Bostik

**Affiliations:** 1 Department of Emergency Medicine and Military General Medicine, Military Faculty of Medicine, University of Defence, 500 01 Hradec Kralove, Czech Republic; ludovit.pudelka@unob.cz; 2 Department of Epidemiology, Military Faculty of Medicine, University of Defence, 500 01 Hradec Kralove, Czech Republic; sylva.janovska@unob.cz (S.J.); pavel.bostik@unob.cz (P.B.); 3 Animal Laboratory, Military Faculty of Medicine, University of Defence, 500 01 Hradec Kralove, Czech Republic; vera.radochova@unob.cz; 4 Department of Medical Microbiology, University Hospital in Hradec Kralove, 500 01 Hradec Kralove, Czech Republic

**Keywords:** honeydew, antibacterial effect, hydrogen peroxide, polyphenolic compounds

## Abstract

An increasing resistance of microbes to antibiotics, the emergence of multidrug-resistant and extremely resistant strains, and the long time needed to develop new antibiotics are driving the search for additional sources of antibacterial agents. The aim of the study was to compare the efficacy of Czech honeys with already available pharmaceutical agents containing medicinal honey, and to perform basic biochemical analysis of Czech samples, including detection of undesirable chemical substances. The results showed strong antibacterial activity of Czech honeydew honeys compared to the control group, especially against G+ pathogens, with an average MIC of 9.44% compared to 17.54%, and comparable activity against G− of 16.48% versus 16.66%. In addition to the strong antibacterial activity, this study confirmed the safety and quality of Czech honeys and helped to select the character of a possible source for in vivo testing and subsequent clinical trials.

## 1. Introduction

Natural products have been used by mankind since time immemorial. Among these products, bee honey has an irreplaceable value. Probably the first written document that mentions the use of honey in wound healing is a papyrus by Edwin Smith, which may have been a war surgeon’s manual, which describes the use of honey in the prevention and treatment of wound infections in ancient Egypt [1]. The antimicrobial activity of honey was first observed in 1892 [2]. In the 20th century, interest in honey and other natural products gave way to the importance of newly discovered antibiotics in developed countries. However, with the emergence of resistant and extremely resistant bacterial species, the use and research of medicinal honey, as with other bee or natural products, is again gaining momentum.

The essence of “liquid gold”, as honey is sometimes called, combines a number (180–200) of different compounds (water, sugars, minerals, vitamins, proteins, phytochemicals, and polyphenolic compounds) with antibacterial properties [3,4]. This composition depends mainly on the bee pasture, and its unique combination may be the reason why the adaptation of microorganisms to honey (e.g., by mutation of genes involved in methylglyoxal detoxification) is very slow or would lead to cross-resistance to antibiotics [5]. Basic factors include the following: (1) Low water activity, which is a scaleless quantity between 0 and 1 that expresses the amount of free, unbound water. It is the free water that microorganisms can use for their growth. Simply stated, the greater the amount of free water, the better the conditions for microbial growth and vice versa. The water activity is usually 0.6, which is sufficient to inhibit osmotolerant yeasts [6,7,8,9]. (2) The presence of polyphenolic compounds, including flavonoids and phenolic acids (nonflavonoids), which are secondary metabolites of flowers and enter honey due to their presence in nectar collected by bees from plants, contributes to the antibacterial effect of honey (nonenzymatic H_2_O_2_ production) [10]. Therefore, bee grazing, which is itself influenced by both the geographical location and season, can significantly affect the phytochemical spectrum and, consequently, the actual antimicrobial effect [10,11,12,13,14]. (3) Hydrogen peroxide is probably one of the main antibacterial factors, which is produced by the aforementioned nonenzymatic route (polyphenolic compounds) or enzymatically via the enzyme glucose oxidase (GOX), which bees add to nectar. Some studies report its production by plants, honey fungi, yeasts, and bacteria. GOX is naturally present in an inactive form and its activity increases with honey dilution, reaching a maximum in honey solutions with a concentration between 30 and 50% [15,16,17,18,19,20,21]. (4) Acidity—the low pH of honey is another factor that contributes to an environment unsuitable for the growth of microorganisms. A large number of micro-organisms need a neutral pH for their growth, but this is not present in honey due to the presence of acids, particularly organic gluconic acid, which is produced by the activity of the enzyme GOX. According to various sources, the average pH of undiluted honey ranges from 3.2 to 4.5 [22]. (5) Honey also contains a large number of proteins, which themselves have antimicrobial activity or at least contribute to the overall antimicrobial activity of honey. One of these is defensin-1, which is found in the hemolymph of bees as part of their immune system. It is also secreted by the hypopharyngeal glands of bees, from where it enters the honey [23]. Studies have shown that defensin-1 is mainly effective against Gram-positive bacteria, but a significant potential against Gram-negative bacteria has been demonstrated as well [24]. In addition to its own antimicrobial effect, it plays an important role in wound healing by stimulating the secretion of matrix metalloproteinase-9 (MMP-9) by keratinocytes [25]. (6) 1,2-dicarbonyls: nonenzymatic reactions between glucose and free amino acid groups can occur in carbohydrate-rich foods when heated or stored for long periods of time. These processes (caramelization, Maillard reaction) can produce the highly reactive compounds mentioned above. The resulting products of these reactions are methylglyoxal and glyoxal, which are essential for the antimicrobial activity of some honeys. Methylglyoxal is a substance that is formed during honey storage by the conversion of dihydroxyacetone in the presence of arginine and lysine, which is abundantly present in the nectar of *Leptospermum* sp. Due to the presence of methylglyoxal, honey retains its antibacterial activity despite inactivation of H_2_O_2_ by catalase, thus demonstrating the nonperoxidase antibacterial activity of honey [26,27,28,29,30,31,32,33]. Today, there are many honeys that have found their way into human medicine. However, their price and quantity are considerable in relation to the need, for example, for the treatment of burns or chronic wounds. Along with environmental and fiscal policies, there is, therefore, an effort to find primarily local sources of honey that could be used as medical-grade honey (MGH). There is no definition of what MGH is, but based on the work of Hermann et al., MGH should meet the following criteria: natural origin, purity, absence of toxic substances and contaminants, sterilization by gamma radiation in accordance with standardized protocols to exclude pathogenic microorganisms, applicability in treatment, fulfilment of physicochemical criteria necessary for use in wound care, and compliance with standardized production and storage methods and all safety regulations [34]. 

However, it is not only the antibacterial properties of honey that are currently the subject of intense research. Thanks to its composition and, in particular, the presence of substances derived from nectar or honeydew, especially from the group of polyphenolic compounds, honey has a number of other properties. Recently, the results of a study have been published describing the inhibition of breast cancer cell migration by honeydew from a conifer-rich area [35], with minimal effect on the function of fibroblasts. On the other hand, honeydew from an oak-rich area showed an inhibitory effect on gastric adenocarcinoma cells, even at low concentrations [36]. The neuroprotective effect of flavonoids in honey has also been demonstrated by reducing neuroinflammation with a reduction in the production of proinflammatory cytokines and ROS [37,38,39]. The use of honey in the treatment of burns, which are characterized by increased ROS production leading to lipid peroxidation and subsequent scarring and contractures, has the same basis as noted [40].

In general, all honeys can be expected to possess antibacterial activity, but there is an effort to select those that have the highest potency. Based on this, we decided to analyze Czech honeys that would meet the same criteria as MGHs and at the same time show maximum antibacterial activity compared to the already available MGHs. Since previous studies [3,41,42] have shown honeydew honeys to be at least as effective as MGH control samples, we decided to focus on honeydew honeys, their qualities, antibacterial activity, and mechanism of action.

## 2. Results

### 2.1. Characteristics of Honeys 

#### 2.1.1. Physicochemical Analysis 

Conductivity was measured to determine the origin of the honeys. In 30 out of 49 samples, a conductivity higher than 80 mS/cm was observed, which, in terms of origin [43], represents honeydew honeys, which are generated by the conversion of honeydew produced by hemipteran phloem-feeding insects such as aphids, mealybugs, whitefly, and psyllids, while the remaining 19 samples showed conductivities lower than 0.8 mS/cm and are therefore nomenclaturally blossom honeys produced by collecting nectar from plants and subsequently converting it into honey.

**Figure 1:**
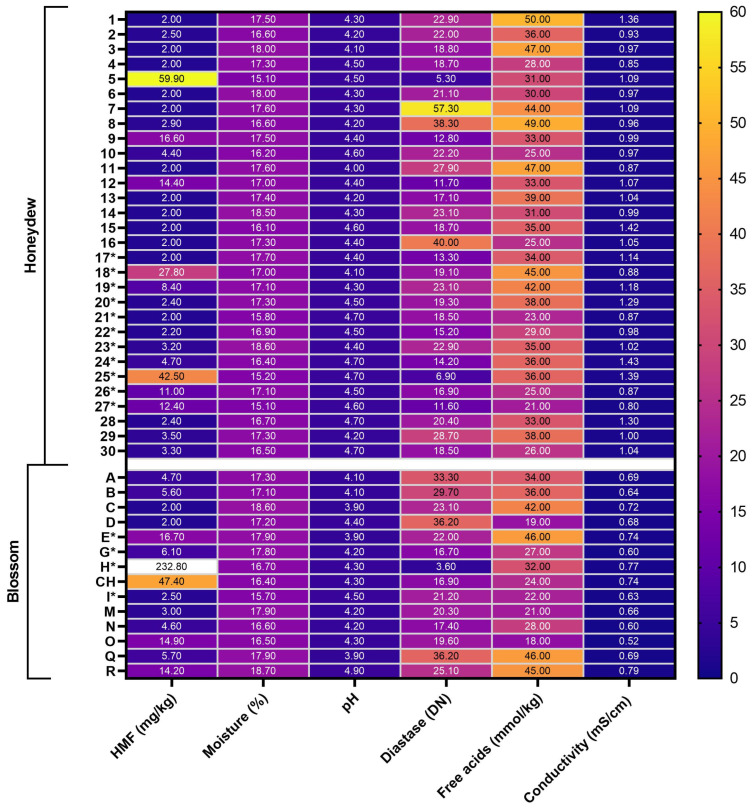
Results of basic physicochemical analysis including determination of HMF (5-hydroxymethylfurfural) mg/kg, moisture—water content g/100 g, pH, diastase activity (Phadebas method) DN, free acids—free acidity mmol/kg, conductivity mS/cm. Samples marked with * come from 2019.

To exclude the influence of honey quality on the main parameters under investigation, hydroxymethylfurfural (HMF) level, water percentage, pH, diastase level, and free acidity were subsequently determined. These results, together with the conductivity results, are presented in Figure 1. 

According to the European directive (110/2001 as amended) [44], the level of HMF must not exceed 40 mg/kg, with exceptions for honey coming from countries or regions with tropical temperatures or honeys with low enzymatic levels. This value was exceeded twice in both groups—honeydew and blossom honeys. These results may be due to poor storage or overheating of the honey during liquefaction. The average value of HMF for the blossom honeys was 25.87 mg/kg, and for the honeydew honeys, 8.28 mg/kg. The average value obtained for the blossom honeys in particular is strongly influenced by the excess level for sample H, which showed the highest value of 232.8 mg/kg among all the samples. This likely indicates a significant heating of the honey during its liquefaction. 

With regards to the water content, the European directive (110/2001 as amended) [44] allows a maximum of 20%, while the Czech directive [45] allows only 18%. This is again an important criterion for the quality of honey shelf life, as the low water content guarantees the stability of the honey and reduces the risk of spoilage mainly due to fermentation processes. All our tested honeys clearly met the European honey quality criteria. Two blossom honeys (18.1–18.7%) and three honeydew honeys (18.5–18.6%) failed to meet the national (Czech) criteria [45], but only by a very narrow margin. 

Although the exact recommended pH value of honey has not yet been determined by the Regulatory Committees, the literature generally indicates the pH range of honey as 3.2 to 4.5, and in some sources 3.52 to 5.13 [46]. This low pH prevents bacterial growth and reduces the risk of spoilage. The pH value of all our samples did not exceed 4.9, with a range of 3.9 to 4.9. 

Free acidity/content of free acids is another parameter, values of which can indicate honey deterioration, similarly to higher pH or water content. The value depends mainly on the quantity of organic acids and inorganic ions such as phosphates, sulphates, and chlorides. European guidelines (EU Directive 110/2001 as amended) [44] allow for a maximum value of 50 mmol/kg [47]. The free acid values for our honeydew honeys ranged from 23 to 50 mmol/kg (mean 35.93 mmol/kg) and 18 to 46 mmol/kg (mean 32.19 mmol/kg) for blossom honeys. 

The diastase activity value, which is given as the diastase number (DN) or Schade or Gothe unit, represents the amount of enzyme that will convert 0.01 g of starch to the prescribed endpoint in one hour at 40 °C under the test conditions, and is set at a minimum value of 8 DN under European legislation (EU Directive 110/2001, as amended) [44]. This value is mainly used on the European market as a parameter of the age/quality of the honey, since its value decreases with age or improper handling of the honey (heating). This is consistent with our results, where the measured value of diastase activity was below the required limit in only three samples (5, 25, H). At the same time, in all these samples, as well as in the CH sample, an above-limit value of 5-hydroxymethylfurfural (HMF) was found, indicating improper storage or processing (overheating) of the honey. Overall, diastase values for honeydew honeys in our samples were in the range of 5.3 to 57.3 °DN (mean 22.5 °DN) and for blossom honeys from 3.6 to 36.2 °DN (mean 21.32 °DN).

#### 2.1.2. Melissopalynological Analysis 

The most frequently represented pollen families in the honeydew honeys were Brassicaceae—present in 93% of the samples, Asteraceae 77%, Salicaceae 67%, Fabaceae 57%, Sapindaceae/Hippocastanoideae 43%, Malvaceae 40%, Boraginaceae 37%, Apiaceae 33%, Rosaceae 33%, Cornaceae 10%, and Polygonaceae 6%. The families Fagaceae and Rhamnaceae were equally represented in 3% of the samples. For blossom honeys, the families were represented in the samples as follows: Brassicaceae in 93% of the samples, Salicaceae in 73%, Fabaceae in 60%, Sapindaceae/Hippocastanoideae and Asteraceae equally in 53%, Boraginaceae in 40%, Malvaceae in 27%, Apiaceae and Rosaceae equally in 20%, and the families Polygonaceae, Balsaminaceae, and Fagaceae were similarly represented in 6% of the samples.

**Figure 2:**
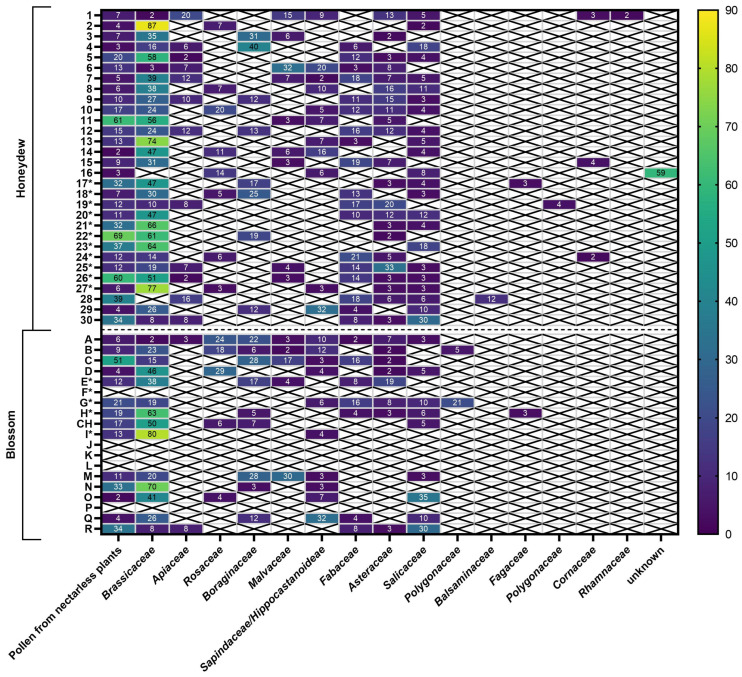
Pollen analysis of honey samples with relative frequency of pollen grains of different plant families (>46% very frequent pollen; 45–16% frequent pollen; 15–3% single pollen; >3% isolated pollen). Samples marked with * come from 2019.

In terms of the number of pollen grains identified in the samples studied, the Brassicaceae family was dominant. With the exception of one sample (No. 20) out of the 44 samples examined, where more than 46% of pollen grains of a particular family were found (Nos. 2, 7, 14, 16, 17, 18, 21, 24, 26, 28, 27, B, D, E, F, G, K), it was the *Brassicaceae* family that was identified. The average percentage of pollen grains of this family in the above samples was 61%, with values ranging from 46 to 87% (see Figure 2).

#### 2.1.3. Presence of Chemical Substances 

In view of the possible future use of honey for medical purposes as medical-grade honey, the samples were also analyzed for the presence of undesirable substances (especially pesticides; the presence of antibiotics was not examined due to their banned use for the treatment of bee colonies in the Czech Republic). Out of all 44 samples, undesirable substances were detected in 9 honeydew samples. In 7 of these samples, substances from the acaricide group were detected (4× Acetamiprid in the range 0.012 to 0.038 mg/kg and 3× Thiacloprid, 0.01–0.072 mg/kg). For the two remaining samples, the fungicide carbendazim was present at a concentration of 0.003 mg/kg. A mixture of three substances was found in one sample—benzyldimethyloctadecylammonium BAC C18 (0.028 mg/kg), benzalkonium chloride (0.067 mg/kg), and cetalkonium chloride (0.039 mg/kg).

Carbendazim (methyl benzimidazol-2-ylcarbamate) metabolized to the bioactive substance benomyl is a broad-spectrum fungicide used worldwide to treat fungal diseases primarily of agricultural crops. However, carbendazim is no longer registered in the EU. The current European maximum residue level (MRL) for carbendazim in honey is 0.05 mg/kg.

Acetamiprid (a neonicotinoid insecticide), used mainly for the protection of agricultural crops such as oilseed rape (vs. *Meligenthes aeneus*), has its MRL set at 0.05 mg/kg in the EU. The same group also includes Thiacloprid, banned on the European market in 2020, whose MRL is 0.2 mg/kg for honey, and which was also used for the protection of agricultural crops such as oilseed rape.

Benzyldimethyloctadecylammonium BAC C18, benzalkonium chloride, and cetalkonium chloride belong to the group of quaternary ammonium compounds, which are mainly used in pharmaceutical and medical applications, as part of preservatives and disinfectants. Their European MRL is 0.1 mg/kg [48].

The analysis for the presence of undesirable chemicals was performed by an accredited laboratory as a paid-for service, and its subcontractors are listed in the Materials and Methods section.

### 2.2. Antibacterial Activity of Honey Samples

**Figure 3:**
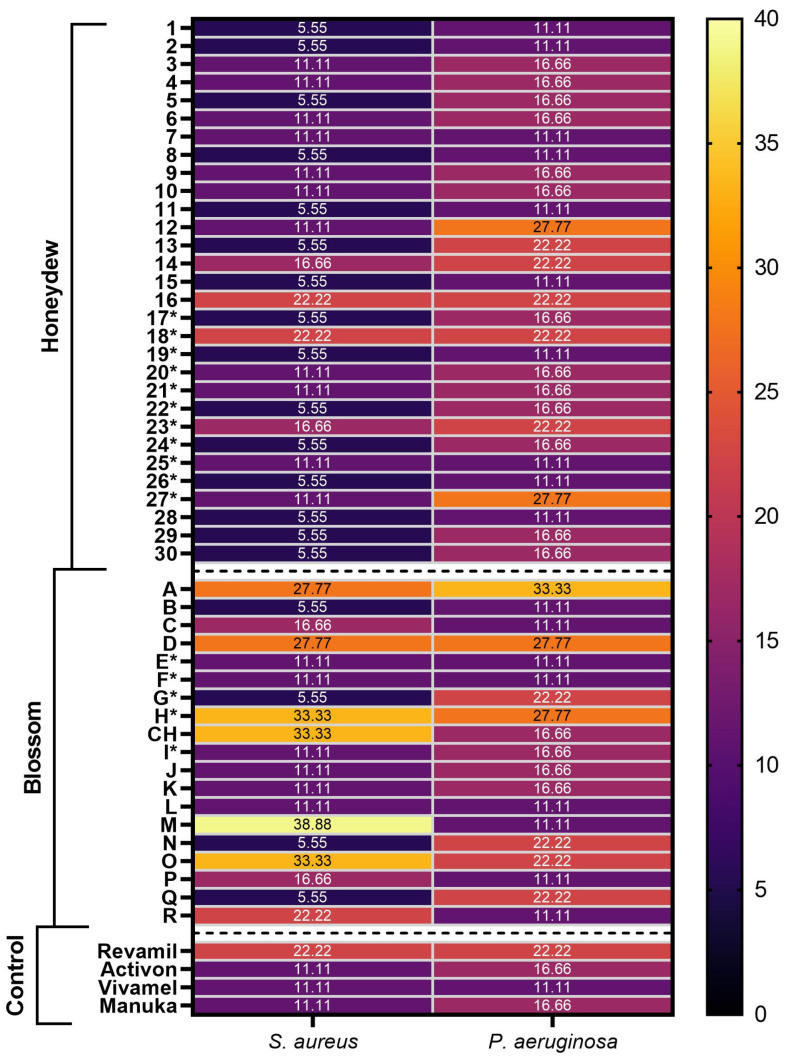
Antibacterial activity of honeydew, blossom, and control samples against bacterial strains of *S. aureus* and *P. aeruginosa*. Antibacterial activity was determined as minimum inhibitory concentration (MIC). MIC was defined as the lowest concentration of honey solution (%) that can inhibit bacterial growth. Samples marked with * come from 2019.

The minimum inhibitory concentrations (MICs) of the honey samples were tested against two bacterial strains representing both G+ and G- bacteria (Figure 3). In addition to the original 44 samples sent abroad for analysis, the MIC test included a further 5 samples that were received later. As controls, medical products purchased from pharmacies with medical-grade honeys (Activon^®^, Vivamel^®^, Revamil^®^) and a sample of manuka honey were included in the study. The MICs of the samples against *S. aureus* (SA) ranged from 5.55% to 22.22% (honey concentration in solution) for the honeydew honeys and 5.55% to 38.88% for the blossom honeys. For the control samples, the range measured was from 11.11% to 22.22%. Thus, the average MIC value for the honeydew honeys was lower, at 9.44%, compared to the average values of 17.83% for the blossom honeys and 17.54% for the control group. Fifteen honeydew samples (1, 2, 5, 8, 11, 13, 15, 17, 19, 22, 24, 26, 28, 29, 30) and four blossom samples (B, G, N, Q) were found to be more effective (5.55%) against *S. aureus* compared to control samples (11.11–22.22%). For the *P. aeruginosa* strain (PSAE), the average MIC values of the samples were very similar. The mean MICs were 16.48% (11.11–27.77%), 17.54% (11.11–33.33%), and 16.66% (11.11–22.22%) for honeydew, blossom, and control samples, respectively. The best MIC value of the control samples was 11.11% (Vivamel^®^). The best honeydew (10 out of 30) and blossom (8 out of 15) samples achieved the same value. 

### 2.3. H_2_O_2_ and Total Polyphenolic Compound Contents in Honey Samples

**Figure 4:**
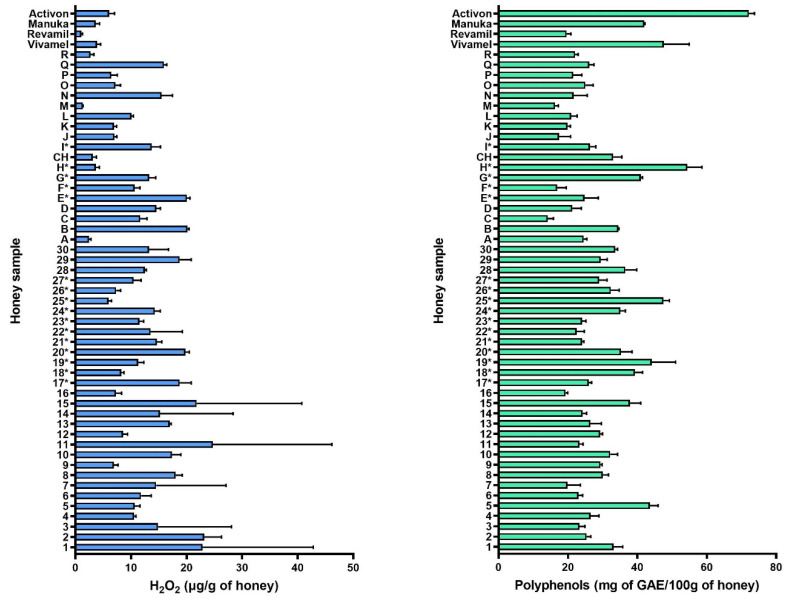
Hydrogen peroxide (H_2_O_2_) and total polyphenolic compounds (TP) in honeydew and blossom honeys. H_2_O_2_ content was measured in honey solution (40% *w*/*w*), immediately after the homogeneity of this solution was reached, without prior incubation. Gallic acid (GAE) was used as TP reference standard compound and results are expressed as GAE equivalents (mg GAE/100 g of honey). Data represent mean values with standard deviation. Samples marked with * come from 2019.

Hydrogen peroxide is one of the most important antibacterial components of honey. It is known to be produced not only by the activity of the enzyme glucose oxidase (GOX) but also nonenzymatically by the contribution of polyphenolic compounds contained in the nectar of plants. In order to assess the importance of the amount of hydrogen peroxide on the overall antibacterial activity, the hydrogen peroxide content of all samples was determined in a 40% honey solution at pH 7.0 immediately after this solution reached homogeneity (Figure 4). The mean H_2_O_2_ value measured was 12.92 µg/g honey. The average value for the control samples was 3.67 (with a range of 1.08–3.89 µg/g honey). The lowest value was measured for the Revamil^®^ sample and the highest value was observed for the Activon^®^ sample. Compared to the control samples, the average level measured for both groups was higher, with 9.84 µg/g honey for the blossom samples and 16.10 µg/g honey for the honeydew samples. The highest value was measured in honey sample number 1 (34.38 µg/g honey), while the lowest value was measured in the control sample Revamil^®^ (1.08 µg/g honey). 

The polyphenol content of all 53 samples was also determined (Figure 4). As mentioned above, polyphenolic compounds may be involved in the formation of H_2_O_2_ and thus contribute to the production of one of the major antibacterial agents. In addition, polyphenolic compounds (phenolic acids and flavonoids) have also been shown to possess intrinsic antimicrobial activity with a broad mechanism of action (inhibition of DNA gyrase, induction of topoisomerase IV-mediated DNA cleavage, cell wall damage with pore formation, etc.) [3,10,41,49,50,51,52,53]. The average content in our honey samples was 28.333 mg GAE/100 g honey. In the control group, the mean was 45.348 mg GAE/100 g honey. The mean value for honeydew honeys was 30.212 mg GAE/100 g honey (range 19.332 to 47.539 mg GAE/100 g honey) and for blossom honeys 25.366 mg GAE/100 g honey (range 34.533 to 14.174 mg GAE/100 g honey). Overall, the highest value was measured for the Activon^®^ sample from the control group (73.289 mg GAE/100 g honey).

### 2.4. Correlations of Parameters

**Figure 5:**
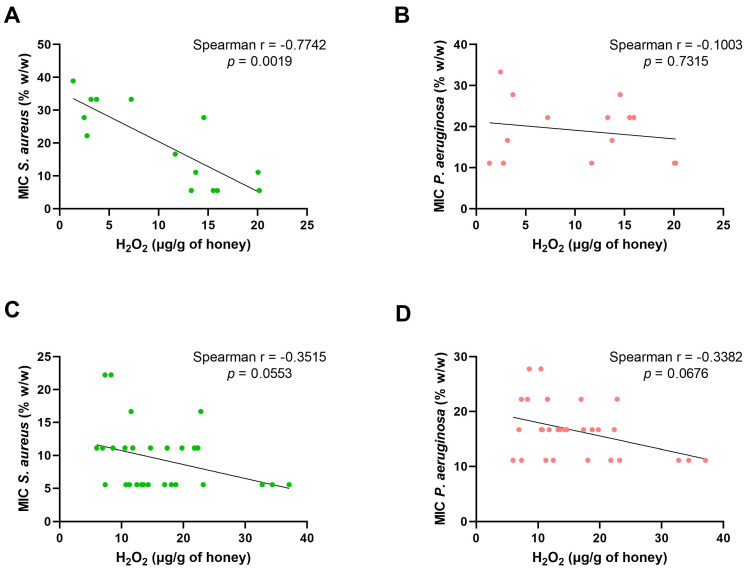
Graphical expression of the relationship between the amount of hydrogen peroxide and the total antibacterial activity (MIC) against both bacterial species *S. aureus* (**A**,**C**) and *P. aeruginosa* (**B**,**D**) in honeydew (**A**,**B**) and blossom (**C**,**D**) honey samples using the nonparametric Spearman’s rank correlation coefficient test with a 95% confidence interval.

As stated in the beginning, most of the honeys were subjected to a very detailed physicochemical analysis, mainly to exclude the potential influence of these parameters, in many cases used to assess the quality of honey, on the antibacterial activity of these samples. To identify possible correlations, Sperman’s rho test was used, the results of which are presented graphically in Figure 5. We investigated the possible correlation between conductivity, which depends mainly on the content of mineral salts and organic acids [54,55], and the amount of free acids and MIC for both strains (SA, PSAE). The data showed a positive correlation between conductivity and the quantity of free acids, but only for blossom honey samples (r = 0.5740, *p* = 0.0343), and not for honeydew honey samples (r = 0.2950, *p* = 0.1135). Furthermore, a Spearman correlation analysis was performed to investigate the relationship between MIC and conductivity, whose value is influenced by minerals and free acids, which may also contribute to the antimicrobial activity of honey through the Fenton reaction or nonenzymatic production of hydrogen peroxide. However, no statistically significant dependence of MIC on the conductivity value for either the honeydew samples (PSAE r = −0.2115, *p* = 0.2619; SA r = −0.2401, *p* = 0.2013) or the blossom samples (PSAE r = −0.1852, *p* = 0.5210; SA r = 0.2840, *p* = 0.3207) was found. To further investigate the importance of polyphenolic compounds in honey and their influence on its antibacterial properties, correlations of factors whose levels could be influenced by the quantity of polyphenolic compounds (conductivity, pH, free acids, and MIC) were subsequently analyzed. However, no statistically significant correlation with MIC for honeydew honeys (SA r = −0.3052, *p* = 0.1010; PSAE r = −0.2786, *p* = 0.1361), or for blossom honeys (SA r = −0.1902, *p* = 0.5111; PSAE r = 0.2257, *p* = 0.4340), was observed. Similarly, no correlation with conductivity was found for blossom honeys (r = −0.0331, *p* = 0.9123). However, a statistically significant moderate correlation was detected for honeydew honeys (r = 0.4339, *p* = 0.0166). No statistically significant correlation was observed between the amount of hydrogen peroxide and polyphenolic compounds, nor in honeydew honey samples (r = −0.2232, *p* = 0.2359), nor in blossom honey samples (r = 0.2, *p* = 0.4924). No correlation between polyphenolic compounds and pH was shown for the blossom honeys (r = 0.1182, *p* = 0.6885), while the value for the honeydew honeys was at the borderline of statistical significance with a weak correlation (r = 0.3569, *p* = 0.0534). The correlation between polyphenolic compounds and free acids was not conclusive for either blossom (r = 0.0286, *p* = 0.9242) or honeydew (r = 0.1553, *p* = 0.4124) samples. The last value that the correlation analysis focused on was the amount of hydrogen peroxide, as one of the two key substances in honey reported so far, which, together with methylglyoxal, is involved in the antibacterial effect. Thus, comparisons of the MIC values against the H_2_O_2_ levels in both honey types for both bacterial strains were calculated. Interestingly, a statistically significant negative correlation between H_2_O_2_ level and MIC in blossom honeys for *S. aureus* (r = −0.7742, *p* = 0.0019), but not for *P. aeruginosa* (r = −0.1003, *p* = 0.7315), was found. For honeydew honeys, the correlations were weak, and on the borderline of statistical significance (*S. aureus* r = −0.3515, *p* = 0.0553, *P. aeruginosa* r = −0.3382, *p* = 0.0676).

## 3. Discussion

The healing properties of honey have been known for thousands of years. Increased antibiotic resistance, the emergence of multidrug-resistant strains, and the search for possible solutions have led to a renaissance of interest in this natural product. Nowadays, there are a number of products that make use of the capabilities of honey (MGHs—medical-grade honeys) and which are used especially in the healing of chronic wounds or, for example, burns. Wider use, particularly in the area of burns, is partly hampered by the price, a significant part of which is transport. This, together with the question of the possible mechanism of action, led us to a study testing Czech honeys. On the basis of previous studies that have shown equal or higher antibacterial potential of honeydew honeys [41,42], we decided to focus on these. 

### 3.1. Quality Parameters

We demonstrated quality and safety of Czech honeys from local producers. Of the 44 samples tested, only 4 exceeded the permitted levels of HMF, and of these 4, the majority were only slightly above the limits. For sample H in particular, it is reasonable to speculate on its liquefaction and subsequent heat damage. This would be consistent with the high HMF and high MIC values for both bacterial strains. However, this expected pattern did not occur for the remaining samples, probably due to a slightly higher HMF value. Sample 5 showed the expected decrease in diastase levels, but its MIC values were among the best. Sample 25 had borderline diastase levels but maintained average MIC values. Sample CH had a normal diastase level, but its MIC values were among the worst, especially for the *S. aureus* strain. The same can be seen from another point of view in samples M, O, 16, and 18, with low HMF values, acceptable diastase values, but below average MIC values.

Moreover, the average HMF value of our blossom samples would be higher than that of the honeydew samples, which is not in agreement with a number of studies dealing with this issue [52,53,54]. On the other hand, the values are more in line with the general assumption that blossom honeys relatively contain a higher proportion of glucose and therefore crystallize faster, depending on the nectar source, leading to more frequent liquefaction during processing, which certainly has an effect on the HMF value of these samples.

Moisture content was exceeded only slightly in samples C, R, 14, and 23, and only when compared to Czech standards. According to the European legislation, even these samples would have easily met the required value of 20%. 

### 3.2. Undesirable Substances—Chemical Compounds

In eight of the samples tested (8, 12, 14, 17, 20, 21, 22, 30), chemicals were detected that should not be present in honey. These were mainly chemical compounds from the group of fungicides and insecticides used mainly in industrial agriculture to protect crops. Paradoxically, however, one sample out of two containing a fungicide (carbendazim) did not come from an apiary that was within the range of bee grazing associated with industrial agriculture. It was, however, in the vicinity of a golf course that uses the fungicide in the protection of golf course turf against earthworms. The insecticides (neonicotinoids) acetamiprid and thiacloprid are both mainly used to protect agricultural crops such as oilseed rape. The use of thiacloprid has been banned in the EU since 2020. However, due to the honey harvesting of our samples that took place in 2020 and 2019, its presence is possible. All the samples in which the insecticide acetamiprid was detected also had a high proportion of pollen grains of Brassicaceae, the family to which oilseed rape belongs, in the range of 47 to 61% of pollen grains per sample. It can therefore be assumed that the contamination of the honey samples came from bees grazing on industrially grown plants such as oilseed rape, the cultivation of which is very intensive in terms of the use of chemicals to protect it. However, it should be noted that all the measured concepts are far below the European limits for the substances in question or their standard detection limits. Nevertheless, it appears that rigorous control of honeys that could be used as medical-grade honeys is essential, as is the choice of the locations of bees whose honey should be collected for these purposes. 

### 3.3. Antibacterial Activity of Samples

As in other studies [42,56,57], our analysis confirmed the effectiveness of local—Czech—honeydew honeys compared to already available medical honeys. The average MIC against *S. aureus* was almost 32% lower in Czech honeydew honeys than in the control group containing MGHs. The situation was less pronounced for *P. aeruginosa*, where the results were almost comparable—the average MIC of the Czech honeydew honeys was 16.48, while that of the control group was 16.66. 

### 3.4. Relationships between Parameters

Subsequent correlation analysis of the results showed no statistically significant relationship between diastase and MIC for either honey type or either bacterial strain. Therefore, it can be assumed that a slight exceedance of the diastase level may not affect the antibacterial capacity, depending on the type of honey.

We demonstrated a statistically significant correlation between conductivity and the quantity of polyphenolic compounds in honeydew honeys (r = 0.4339, *p* = 0.0166), which is consistent with the above text and demonstrates the possible influence on the resulting conductivity of the presence of organic acids belonging to the group of polyphenolic compounds. This correlation was not proven in the case of blossom honeys. On the other hand, in contrast to honeydew samples, we found a positive correlation between free acidity and conductivity values for blossom honeys (r = 0.5740, r = 0.0343), as did Balos et al. for meadow and polyfloral honeys [58]. In contrast, for pH, the correlation with phenolic compounds is weaker and also on the borderline of statistical significance (r = 0.3569, *p* = 0.0534). This is again consistent with the mechanism described above and the more significant influence of mineral compounds on the resultant value. For the blossom honeys, as for the previous value, this correlation was not observed. 

### 3.5. Mechanism of Antibacterial Activity of Honey

Another question we were attempting to answer was the possible mechanism of action of Czech honeys. A number of previous studies suggest that the unique antibacterial properties of honey are the result of the interplay of a number of factors (pH, low water activity, high sugar content, presence of proteins, H_2_O_2_ formation, and a number of other compounds such as methylglyoxal and polyphenolic compounds). This is also documented by a number of studies showing that the activity of one factor alone is not sufficient to achieve antibacterial capabilities and that it is, as mentioned above, a complex effect of several factors on the bacterial cell [3,59,60]. 

Leaving aside the antibacterial effect of honeys based on the presence of methylglyoxal, which, according to some studies, can probably inhibit other antibacterial mechanisms (low levels of GOX in manuka honeys) [28], the antibacterial activity is based on H_2_O_2_. Considering the results of analyses of honeys from neighboring countries, it is very difficult to assume that single-species honeys containing a unique substance similar to methylglyoxal are present in the Czech Republic and, as in other countries, their activity will be mainly the result of the cooperation of H_2_O_2_ and polyphenolic compounds. We based our hypothesis on this assumption and focused on these two factors. 

It is known that the formation of hydrogen peroxide in honey takes place, among other mechanisms, by an enzymatic reaction mediated by the enzyme GOX. On the other hand, no correlation was found between the amount of GOX and H_2_O_2_ [3,41]. Interestingly, the level of this enzyme produced in the pharyngeal glands of the honey bee is controlled/dependent not only on the age of the bee and its roles in the community [61], but probably also on the composition of the honey bee forage [60] and the genetic diversity of the honey bee [62]. In the interspecies comparison of honeys, honeydew honeys produced higher amounts of H_2_O_2_ than blossom honeys, which was confirmed in our study, where the mean H_2_O_2_ value of blossom honeys was lower than that of honeydew honeys (10.40 µg/g honey vs. 16.10 µg/g honey, respectively), which is probably related to the presence of polyphenolic compounds in honeydew honeys [41,42,60,62]. Another explanation may lie in the presence of the enzyme catalase. Catalase is abundant mainly in flower pollen, and much less in nectar. Nevertheless, it can be considered that in certain situations, such as the availability of intensive monofloral bee pasture, the type of which (pollen, nectar) contains high levels of catalase, the resulting production and, hence, the overall antibacterial activity may be more significantly affected. This is particularly the case if the flower constancy aspect is also taken into account. This hypothesis is also supported by the knowledge of the origin of honeydew honey, the source of which is predominantly not nectar but honeydew, as we wrote earlier [63,64]. 

In our study we showed a statistically significant negative correlation (r = −0.7742, *p* = 0.0019) between the amount of hydrogen peroxide and MIC in blossom honeys for G+ (*S. aureus*) bacteria, similarly to other authors [3]. For honeydew honeys, the correlation was weaker and, moreover, at the borderline of statistical significance (r = −0.3515, *p* = 0.0553). For G− bacteria, represented by *P. aeruginosa*, we did not demonstrate the above-described correlation for either type of honey.

The second option of hydrogen peroxide formation is the nonenzymatic pathway, in which polyphenolic compounds play an important role due to their oxidation–reduction potential. An example is the flavonoid quercetin, which, according to various studies, is one of the most common in honeys, as are chrysin, pinocembrin, galangin from propolis [65], or ferulic acid and kaempherol [41,66,67]. The oxidation of quercetin to quinone (an oxidized derivative of aromatic compounds) produces, among other things, H_2_O_2_ [68].

The relationship between MIC for *S. aureus*/*P. aeruginosa* and the content of polyphenolic compounds that has been shown in blossom honeys [3] was not confirmed in our sample of honeydew or blossom honeys. Thus, the resulting effects of the two main factors can be expected to be much more complex. Honeydew honeys are known to contain higher mineral content than blossom honeys, the amount and type of which depend mainly on the type of bee foraging, its geographical location, and possible secondary environmental contamination [69]. In relation to the previous text, this is mainly due to the presence of elements with oxidation–reduction potential such as Fe and Cu. It is in the presence of these elements that the abovementioned reactions of polyphenolic compounds take place. Analysis of Polish blossom honeys (acacia, buckwheat, linden, oilseed rape, raspberry, mixed) showed the highest abundance of Fe together with Mn and Zn in buckwheat honeys and a negative correlation between copper and, on the other hand, sodium and calcium [69]. A Swiss study showed a high Fe and Cu content in honeydew honey (electrical conductivity 97 mS/cm), probably with a predominant pollen content of fir (designated as fir in the study) [70]. A final clue is the formation of reactive oxygen species in the Fenton reaction requiring the presence of elements with high redox potential such as the elements iron and copper. It is in the presence of these elements and hydrogen peroxide that superoxide and hydroxyl radicals are formed [71]. The interdependence was presented in a study by Rane (2021) [72], where inactivation of hydrogen peroxide by catalase led to inhibition of polyphenol autooxidation. At the same time, it confirmed the assumption that it is the combination of these two factors that is responsible for DNA degradation, as opposed to H_2_O_2_ alone [72]. Indeed, as Brudzynski et al. report in their article, H_2_O_2_ is a substrate for hydroxyl radical formation [73]. The above assumption is then indirectly confirmed by the results of our study and the study of Bucekova et al., where honey samples with the highest H_2_O_2_ values do not automatically have the lowest MIC values [74]. 

### 3.6. Holy Grail

Based both on the data presented above and previous studies, it can thus be assumed that the imaginary holy grail should be honey with a high content of polyphenolic compounds and sufficient minerals. The data in this study show that such criteria are best fulfilled by honeydew honeys. This hypothesis is supported by studies showing a correlation between honey color and mineral and polyphenol content [21,75,76]. Thus, these honeys should likely originate in areas with honeydew sources and plants that are rich in polyphenolic compounds contained in nectar/pollen (heather, chestnut, buckwheat, fir). However, at present, the authors are not aware of any study that answers the above hypothesis of the complex relationship between the amount and nature of minerals, honey color, quantity of phenolic compounds, pollen analysis, and H_2_O_2_ content in relation to overall antibacterial activity.

## 4. Material and Methods

### 4.1. Honey Samples

A total of 49 honey samples from local producers (33 from 2020 and 16 marked with * in the graphs from 2019) were included in the study. The control group consisted of 3 commercial products containing medical-grade honey (MGH) purchased on the market: Revamil^®^ (Rhenen, The Netherlands), Activon^®^ (Nottingham, UK), Vivamel^®^ (Domžale, The Republic of Slovenia), and 1 package of Manuka Honey MGO 550+ (Manuka Health NZ Ltd., JMH12560, produced 13 August 2019 (Auckland, New Zealand)).

### 4.2. Physical and Biochemical Analysis of Honeys

Forty-four samples were sent for complex analysis to the Eurofins Food Integrity Control Services GmbH laboratory (Ritterhude, Germany) to determine the quality and origin of individual honey samples and their possible comparison or the influence of these factors on the main parameter under investigation—antimicrobial efficacy. The analysis included microscopic analysis (microscopic examination of pollen—500 pollen grains counted, 30 species identified, starch and yeast content, number of honeydew particles, pollen from nectarless plants, percentage of individual pollen grains: >46%, 45–16%, 15%–3%, >3%), physicochemical analysis (electrical conductivity at 20 °C, free acids, diastase activity, pH value, moisture, 5-hydroxymethylfurfural), analysis of undesirable chemical substances—reporting limit in mg/kg (acaricides 0.2–0.002, organophosphorus pesticides 0.01–0.05, organochlorine pesticides, pyrethroids 0.001–0.05). According to the protocol, the analysis consisted of the following methods: gas chromatography with conventional (flame photometric detector—FDP and electron capture detector—ECD) and mass-selective detectors (mass detector—MS), and tandem mass spectrometry—MS/MS. MS/MS was used to detect acaricide residues. GC-ECD was used to detect organochlorine pesticides and pyrethroids, and GC-FDP to detect organophosphorus pesticides. Pesticide screening was then carried out using a multimethod approach for the determination of pesticide residues using GC and liquid chromatography-based analysis after acetonitrile extraction/partitioning and clean-up by dispersive SPE—modular QuEChERS method. QuEChERS is an acronym for quick, easy, cheap, effective, rugged, and safe. 

### 4.3. Bacterial Strains and Culture Conditions

The bacterial strains of *Staphylococcus aureus* (CCM 4223) and *Pseudomonas aeruginosa* (CCM 1960) were obtained from the Czech collection of microorganisms. Strains were cultured on Nutrient agar (Merck, Czech Republic). Bacterial stocks for cryopreservation were prepared on porous beads (ITEST, Hradec Kralove, Czech Republic). For each experiment, fresh bacterial culture was prepared as follows. The porous bead with appropriate bacteria was inoculated onto the agar plate. Culture was performed under aerobic conditions at 37 °C for 24 h. 

### 4.4. Determination of H_2_O_2_ Content

The content of H_2_O_2_ in honey samples was determined using a modified protocol by Megazyme GOX assay kit (Megazyme International Ireland, Wicklow, Ireland) as published [3]. Briefly, the dilution range of 9.8 to 312.5 µM of H_2_O_2_ was prepared as calibration standard. Then, 40% (*w*/*w*) honey solutions were prepared in 0.1 M potassium phosphate buffer (pH 7.0) and immediately measured. For the blank control, the sample or H_2_O_2_ standard was substituted with distilled water. Each sample was tested at least in three replicates. The absorbance was then measured at 510 nm using a Synergy H1 microplate reader, and data were analyzed using Gene5 (version 3.05.11, Biotek, Winooski, VT, USA) software. 

### 4.5. Determination of Polyphenolic Content

The content of polyphenolic compounds was determined from 20% (*w*/*v*) honey samples using Folin–Ciocalteau Phenolic Content Quantification Assay Kit (BioQuochem, Oviedo, Spain) according to the manufacturer’s instructions. The absorbance was measured at 700 nm using a Synergy H1 (Biotek, Winooski, VT, USA) microplate reader, and the polyphenolic content was calculated using Gene5 (version 3.05.11, Biotek Winooski, VT, USA) software. 

### 4.6. In Vitro Antimicrobial Testing of Honey Samples

The evaluation of antimicrobial activities of tested honey samples was performed using a microdilution broth method. Briefly, dilutions of each honey were prepared with a Mueller–Hinton broth resulting in final percentage concentrations of 50, 44, 38, 33, 27, 22, 16, 11, 5.5, 2.75, 1.38, and 0 (*w*/*w*). Subsequently, the bacterial inoculum was prepared from overnight bacterial culture in a saline solution to a final concentration of 10^6^ colony forming units/mL. Afterward, 90 µL of each diluted honey sample was applied into the wells and inoculated with 10 µL of bacterial suspension. A positive growth control consisting of tested bacteria in broth and a negative sterility control consisting of sterile broth were included for each assay. Each testing was performed at least in three replicates. The minimal inhibitory concentration (MIC) was determined after incubation at 37 °C for 24 h. The MIC was defined as the lowest concentration of honey sample in which no visible bacterial growth was detected.

### 4.7. Data Analysis

GraphPad Prism 9 software (version 9.40, GraphPad Software Inc., San Diego, CA, USA) was utilized for graphical outputs and basic statistical evaluation. Normality evaluation was performed using the Anderson–Darling test and Shapiro–Wilk test. For the correlation analyses, the nonparametric Spearman’s rank correlation coefficient test with a 95% confidence interval was used.

## 5. Conclusions

In this study, we compared the antibacterial efficacy of Czech honeys with three representatives of commercially available products containing medicinal honey (Revamil^®^, Activon^®^, Vivamel^®^) and with Manuka Honey MGO 550+ pack (Manuka Health NZ Ltd.). We demonstrated their safety, quality, and efficacy against both G+ and G− pathogens and, based on the results and comparison with published sources, we defined the type of honey that should meet our requirements for a local source of medical-grade honey. However, a series of further analyses are needed to confirm our hypothesis and the possibility of testing this source in vivo and in subsequent clinical trials. 

## Data Availability

The original contributions presented in the study are included in the article, further inquiries can be directed to the corresponding author.
